# An intelligent spraying system for weeds in wheat fields based on a dynamic model of droplets impacting wheat leaves

**DOI:** 10.3389/fpls.2024.1420649

**Published:** 2024-06-14

**Authors:** Qi Xie, Minghan Song, Tong Wen, Weixing Cao, Yan Zhu, Jun Ni

**Affiliations:** ^1^ College of Agriculture, Nanjing Agricultural University, Nanjing, China; ^2^ National Engineering and Technology Center for Information Agriculture (NETCIA), Nanjing, China; ^3^ Engineering Research Center of Smart Agriculture, Ministry of Education, Nanjing, China; ^4^ Collaborative Innovation Center for Modern Crop Production Co-Sponsored by Province and Ministry, Nanjing, China

**Keywords:** weed-herbicide interaction, mathematical model, dynamic simulation, application quality, precision herbicide application

## Abstract

**Introduction:**

Targeted herbicide application refers to precise application of herbicides in weed-infested areas according to the location and density of farmland weeds. At present, targeted herbicide application in wheat fields generally faces problems including the low herbicide adhesion rate, leading to omission and excessive loss of herbicides.

**Methods:**

To solve these problems, changes in the impact force of herbicide and the weed leaves in the operation process of a spraying system were studied from the interaction between weeds and herbicides applied. A dynamic model of weed leaves was established. On this basis, the research indicated that the herbicide adhesion rate is highest under spraying pressure of 0.4 MPa and flow rate of 0.011 kg/s when the spray height is 300 mm. To study the dynamic deformation of weed leaves and the distribution of liquid herbicides in the external flow field under weed-herbicide interaction, a dynamic simulation model of herbicide application was built using the finite element method.

**Results and Discussion:**

The results show that when the spray height is 300 mm, the maximum weed leaf deformation index (LDI) is 0.43 and the velocity in the external flow field is 0 m/s under spraying pressure of 0.4 MPa and flow rate of 0.011 kg/s. This finding indicates that the herbicide is not splashed elsewhere and the turbulence intensity in the weed area is 2%, implying steady flow of the herbicide, most of which can be retained on weed leaves. Field test results of application quality of the herbicide show that the maximum LDI is 0.41 and the coverage of the herbicide in the sheltered area below the leaves is 19.02% when the spraying pressure is 0.4 MPa, flow rate is 0.011 kg/s, and spray height is 300 mm. This solves the problem of a low rate of utilization of herbicides because the herbicide passes through weed plants, and achieves the precision herbicide application in wheat fields.

## 1 Introduction

Wheat is the third most important food crop in the world and feeds 60% of the global population (Liu et al., 2016). Weeds compete with wheat for water, fertilizers, and nutrients, encroach the overground and underground spaces, and interfere wheat growth, so they have become one of the main factors that limit the wheat yield and quality (Shiferaw et al., 2013). Wheat is generally sown in drill, of which the intra-row weed control is one of the most important weed control tasks. At present, intra-row weeds are mainly controlled by spraying lots of herbicides in rows. However, such an extensive mode not only is costly and inefficient but also may pollute the environment. According to the Agricultural Green Development Report of China in 2020, the effective rate of utilization of herbicides in China is only 40.6%, sparking concern about food security and groundwater pollution by herbicides (Zhu et al., 2020). Therefore, it is necessary to improve the precision of herbicide application.

Targeted herbicide application involves precisely applying herbicides in weed-infested areas according to the location and density of farmland weeds; it has become the main method of herbicide application in farmland (Loghavi and Behzadi Mackvandi, 2008). Shearer et al. (Shearer and Jones, 1991) developed and tested a selective weeder carrying opto-sensors, which can detect green weed plants and spray herbicides only onto weeds, which saves 15% of herbicides while ensuring the control effect. Tian et al. (Tian et al., 1999) developed an automatic spraying system guided by a real-time machine vision system, which can control each nozzle independently according to inter-row weed information of each crop, thus reducing the amount of herbicides used. However, stem and leaf treating agents are generally adopted to remove weeds after wheat germinates. Such herbicides mainly play their roles by being absorbed by stems and leaves and are lost mainly due to flowing into soil rather than being retained by weed leaves. This results in the excessive loss of herbicides, failure in effective absorption by weeds, and the low herbicide rate of utilization (Li et al., 2018). Therefore, the research focus on precision herbicide application in wheat fields is designed not only to achieve targeted spraying of herbicides but also to ensure coverage over weed leaves.

Existing research has shown that adjusting the spraying pressure (flow rate) can change the herbicide spray coverage over weeds (Guler et al., 2007). Nordby et al. (Nordby and Skuterud, 1974) explored the influence of nozzle pressure on herbicide spray drift, and results show that as the pressure is increased from 0.2 MPa~0.5 MPa to 1 MPa, the herbicide spray drift enlarges from 1%~4% to 2%~9%. Nuyttens et al. (Nuyttens et al., 2007) experimentally compared the herbicide spray deposition when the spraying pressure is 0.2 MPa~0.4 MPa and the nozzle height is 0.3 m~0.75 m; statistical results show that the herbicide spray drift reduces by 40.1% as the nozzle height is reduced by 0.2 m, and it decreases by 43.1% as the spraying pressure is decreased from 0.3 MPa to 0.2 MPa. However, these studies do not set plants in the tests but only evaluate the influences of working parameters on the nozzle performance by testing the herbicide spray drift while ignoring the bearing capacity of leaves for herbicide spray. As a result, most herbicides flow into the soil, thus leading to a low rate of utilization and waste.

**Figure 1:**
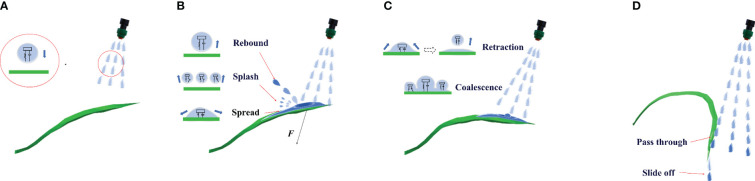
The weed–herbicide interaction process [**(A)** Targeted movement; **(B)** Contact and diffusion; **(C)** Continuous coverage; **(D)** Strong impact and drainage].

In the herbicide application process, the herbicide solution is sprayed with a certain pressure from the herbicide application device and then reaches the weeds. Due to the surface tension and adhesion, herbicide spray adheres to weed leaves. The herbicide application process is illustrated in **Figure 1**, which can be divided into the following stages:

Targeted movement: the herbicide is atomized at the nozzle outlet and then sprayed. In the movement process toward the target, the discrete herbicide spray is under joint influence of multiple forces, including the gravity and surface tension, finally forming stable spherical droplets (Hai, 2007) from the initial irregular shape after repeated stretching and contraction. These droplets have oblique projectile motion under the spraying pressure, and the spraying pressure is directly proportional to the velocity of droplets. A higher velocity of droplets exerts greater impact force on the weed leaves. In this stage, the droplets have not come into contact with the weeds, so the weed leaves remain in their original state and are undeformed.

Contact and diffusion: after reaching the weeds, droplets impact leaf walls. When the generated impact force is stronger than the buckling resistance of leaves, the leaf angle of weeds gradually enlarges. In the process, the droplets can be analyzed by being approximated as a spring mass system (Xu et al., 1998): some droplets flow freely on the surface, the spring system is compressed, and droplets spread on the leaves, whereas some droplets rebound because the elastic force is stronger than the adhesion after collision with leaves (Mundo et al., 1995; Pasandideh Fard et al., 1996). Due to the damping effect, the rebound velocity is lower than the injection velocity. Some droplets show mass displacement exceeding the strength limit of springs after impacting leaves and therefore are splashed (Qun, 2007). In the stage, when the impact frequency of droplets is close to the intrinsic frequency of leaves, the leaves are vibrated slightly and the amount of retained herbicide decreases temporarily.

Continuous coverage: the velocity and direction of rebounded and splashed droplets as well as the particle size of broken droplets are not fixed. Some secondary deposited droplets strike leaves together with other droplets, so that leaves are deformed under the impact force. As the leaf angle gradually increases, the amount of retained herbicide also increases. Droplets spreading in the previous stage retract due to the surface tension after reaching the maximum spread area. In the process, droplets have friction with the leaf surface and therefore lose some energy. The retracted droplets rebound in a small range and coalesce with other droplets deposited on the leaves to form a stable liquid film by adhesion. When the deformed leaves are parallel to the ground, it is defined as the critical leaf deformation in the stage. Under the condition, the amount of retained herbicide reaches the maximum and herbicide in the liquid film constantly infiltrates in the leaf tissues and damages weeds, contributing to a high rate of utilization of herbicide.

Strong impact and drainage: if droplets retain significant amounts of kinetic energy after the previous stage, those leaves aligned parallel to the ground may be further deformed downward. Under that condition, leaves enter the strong impact and drainage stage, during which the herbicide is not retained by weed leaves any longer but it passes through leaves and flows into soil, thus leading to waste. The herbicide spray deposited on leaves gradually slides off to the soil due to the action of gravity. The larger the leaf angle, the more herbicide is lost.

To solve the above problem, the deformation of weed leaves under impact force during herbicide application was explored through analysis of the interaction between weeds and sprayed herbicides. A dynamic model of weed leaves was established, and parameters including the appropriate spraying pressure and flow rate of the spraying system were determined, to improve the herbicide retention rate on weed leaves and realize precision application of herbicides in wheat fields.

## 2 Materials and methods

### 2.1 Dynamic model of weeds in the weed–herbicide interaction process

In the weed–herbicide interaction, the herbicide is atomized into droplets with a certain initial speed under the spraying pressure. These droplets have oblique projectile motion toward weed plants and exert impact force on the leaves after reaching weed positions. When the impact force is lower than or approximate to the buckling resistance of leaves, the weed leaves remain in their initial state. If the impact force exceeds the buckling resistance, the leaves are deformed, the amount of retained herbicide increases, and droplets spread, rebound, and splash on leaf walls in the impact process. If the impact frequency of rebounded droplets and other droplets is similar to the intrinsic frequency of weed leaves, “frequency locking” occurs, accompanied by small-amplitude vibrations (Gosselin and de Langre, 2009). Under the condition, the amount of herbicide reduces slightly. As the rebounded herbicide stabilizes on leaves, the vibration amplitude of weed leaves reduces and the leaves are further deformed downward. At the moment when the deformed leaves are parallel to the ground, the amount of retained herbicide reaches the maximum and deposited herbicide spray constantly infiltrates in leaf tissues to damage the weeds, which contributes to a high herbicide rate of utilization. If the impact force of droplets on leaves is greater than the buckling resistance of leaves, the leaves are more deformed than when they are parallel to the ground. They then enter the strong impact and drainage stage, when the herbicide passes through leaves and reaches the soil, which leads to waste of the herbicide. Under action of droplets, the leaves have attenuation vibration and swing periodically, and their maximum deformation is determined by the magnitude of impact force. The weed–herbicide interaction process shows that the spraying pressure and flow rate can change the impact force of droplets on weed leaves, thus determining the deformation of weed leaves and the herbicide rate of utilization. Therefore, it needs to determine appropriate operating parameters of the spraying system and improve the quality of application of herbicides for weed control.

**Figure 2:**
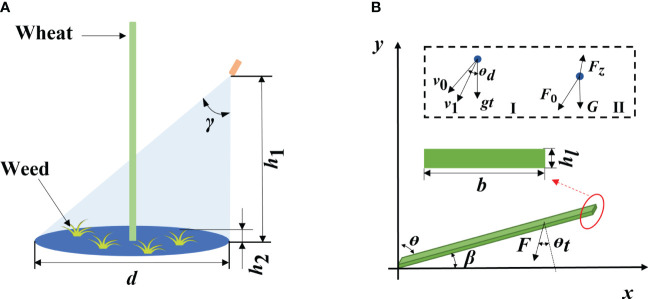
Nozzle parameters and force analysis of weeds [**(A)** Nozzle parameters; **(B)** Force analysis of weeds, in which I is the motion analysis and II is the force analysis of discretized herbicide spray].


In the weeding process, the nozzle was fixed in the middle between wheat rows. The parameters and herbicide application range of the nozzle are shown in **Figure 2A**. The herbicide is discretized at the nozzle, sprayed out at the initial velocity of ${\text{v}}_{0}$ under the spraying pressure. Afterward, the herbicide spray reaches the weed position that is $l_{w}$ from the nozzle via oblique projectile motion. Under these conditions, the discretized herbicide spray reaches the weed leaves at velocity ${\text{v}}_{1}$ . The specific motion analysis is shown in **Figure 2B**I. The calculation formula is as shown in **Equations (1**, **2)**.

(1) $${\text{v}}_{1}=\sqrt{{\text{v}}_{0}^{2}{\text{sin}}^{2}{\text{θ}}_{\text{d}}{\text{+(}v}_{0}{\text{cos}\theta }_{\text{d}}{\text{+gt)}}^{2}}$$

(2) $${\text{l}}_{\text{w}}=\sqrt{{\text{v}}_{0}^{2}{\text{t}}^{2}{\text{sin}}^{2}{\text{θ}}_{\text{d}}{\text{+(}v}_{0}{t\text{cos}\theta }_{\text{d}}+\frac{1}{2}{\text{gt}}^{2}{)}^{2}}$$

where $\theta_{d}$ is the tilt angle of the nozzle (°); t denotes the time taken by the discretized herbicide spray from departing the nozzle to reaching the leaves (s); and g is the gravitational acceleration (m/s²).

The leaves are approximated as rectangles, so they can be regarded as cantilever beams with a rectangular section (Sterling et al., 2023). Taking the root of weed leaves as the origin, the plane parallel to the soil as the x-axis, the growth direction of leaves as the positive direction of the x-axis, and the growth direction of plants as the positive direction of the y-axis, a coordinate system of weed leaves is established. The force on weeds in the herbicide application process is illustrated in **Figure 2B**.

After reaching the leaves, the discretized herbicide spray produces the impact force $F_{0}$ (**Figure 2B**II), which is calculated using the following formula (Imeson et al., 1981; Soto et al., 2014):


(3) $${\text{F}}_{0}=\frac{{\text{πρRv}}_{1}^{2}\text{V}}{2}$$

where *R* is the particle radius of the discretized herbicide spray (m); *V* is the volume of discretized herbicide spray ( ${\text{m}}^{3});\text{ and }\rho$ is the density of the herbicide (kg/m³).

According to the mechanics of materials, the calculation method is as shown in **Equation (4)**.


(4) $$\text{EI}\frac{{\text{d}}^{2}\text{y}}{{\text{dx}}^{2}}\text{=M(x)}$$

where *E* is the elastic modulus of leaves (MPa); *I* is the cross-sectional moment of inertia ( $\text{I=}\frac{{\text{bh}}_{\text{l}}^{3}}{12}$ ); ${\text{h}}_{\text{l}}$ represents the thickness of weed leaves (mm); *b* represents the maximum width of weed leaves (mm); and M(x) is the bending moment of cross sections of leaves.

In the natural state, the bending moment of leaves under herbicide impact force is expressed as **Equation (5)**.


(5) $$\text{M}\mathrm{(x)=EI}\frac{{\text{d}}^{2}\text{y}}{{\text{d}x}^{2}}=\int_{0}^{\text{l}}\int_{0}^{\text{b}}\frac{{\text{F}}_{0}\text{sin}\theta {\text{x}}^{2}\text{cos}\theta_{\text{(t)}}\mathrm{+G}\text{cos}\beta }{{2\text{Acos}}^{2}\text{θ}}\text{d}b\text{d}l$$

where θ is the angle between weed leaves and stem (°) and $\theta_{\text{(t)}}$ is the angle between discretized herbicide spray and normal direction of leaves (°); therein, θ=90-β where β is the leaf angle (°). l is the length of weed leaves (mm), and *G* is the self-weight (gravitational) force on the discretized herbicide spray (N).

It is calculated that


(6) $$\text{y=}\int_{0}^{\text{l}}\int_{0}^{\text{b}}\frac{{2\text{F}}_{0}\text{sin}\theta {\text{x}}^{4}\text{cos}\theta_{\text{(t)}}{\mathrm{+G}\text{cos}\mathrm{\beta x}}^{2}}{{48\mathrm{EIA}\text{cos}}^{2}\theta }{\text{d}b\text{d}\mathrm{l+C}}_{1}{\text{x+C}}_{2}$$

where A is the area of weed leaves ${\text{(mm}}^{2}{\text{), A=bl; C}}_{1}{\text{, C}}_{2}$ represent coefficients of the deflection equation of leaves.

When *x* = 0, according to the boundary conditions of the coordinate system, $y=0,y^{'}=\theta$ . By using Equation 6, it is obtained that ${\text{C}}_{1}=\theta {\text{, C}}_{2}=0$ . According to Equation 3, the mathematical model of deflection of weed leaves is


(7) y=∫0l∫0bπρv12RVsinθx4cosθ(t)+Gcosβ4Eb2lhl  3cos2θdbdl+θx

Analysis of the mechanism underlying the weed–herbicide interaction reveals that when the deflection of leaves is smaller than that when the leaves are parallel to the ground, the herbicide spray can be retained by leaves, thus reaching the goal of weed removal. *In-situ* measurement showed that *l* = 35 mm, β=33.22° , *b* = 15 mm, and ${\text{h}}_{\text{l}}$ = 0.4 mm. It is then calculated that θ=56.788° . Previous research shows that *E* = 272.5 MPa (Cui et al., 2023) and *R* = 1 mm (Qiu et al., 2022). Aqueous herbicide is used in practice such that *ρ* = 1,000 kg/m³ and *G* = 0.0003 N (Lauderbaugh and Holder, 2021). The row spacing of wheat is generally approximately 200 mm (Saha et al., 2021), so the spray range (*d*) of the nozzle on both sides of the crop plant is set to 200 mm, and the cone angle *γ* is calculated using the following **Equation (8)**:


(8) $$\gamma \text{=arctan}\frac{\text{d}}{{\text{h}}_{1}}$$

where ${\text{h}}_{1}$ is the ground clearance of the nozzle (mm); it is calculated that *γ* = 33.6902°, which is rounded to 35°, then $\theta_{\text{(t)}}\;$ ∈ (33.22°, 68.22°).

The ground clearance of the nozzle directly affects the weed control effect in the herbicide application process: if the ground clearance is too large, the herbicide spray may not reach the weeds due to being sheltered by wheat leaves; if the ground clearance is too small, the herbicide is sprayed onto the soil and thus leads to a low rate of utilization. Therefore, the ground clearance of the nozzle should be the height of weeds. The statistical analysis on the weed and wheat heights shows that the two differ greatly and the weed height is generally lower than 300 mm (Xu et al., 2020). Considering this, ${\text{h}}_{1}$ is set to 300 mm. Then, ${\text{l}}_{\text{w}}$ is calculated to be 300 mm~366 mm.

According to the Bernoulli equation, the relationship between the spraying pressure and the velocity of discretized herbicide spray is


(9) Pρg=v0  22g

The relationship between the flow rate and spraying pressure of the nozzle is approximated as follows:


(10) $${\text{Q=}C}_{\text{d}}{\text{A}}_{1}\sqrt{2\text{Δ}\mathrm{P\rho}}$$

where Q is the flow rate of herbicide (kg/s); ${\text{A}}_{1}$ is the area of the nozzle outlet ( m2), A1=πR1  2; R1 represents the radius of the nozzle outlet and is valued as ${\text{R}}_{1}=0.0005{\text{ m; }C}_{\text{d}}$ is the flow coefficient, which is generally between 0.6 and 0.7 and in the research, ${\text{C}}_{\text{d}}=0.6\text{; and Δ}P$ is the pressure difference (Pa).

By using Equations 7, 9, and 10, the spraying pressure of the nozzle is 0.383 MPa ≤  P  ≤ 0.389 MPa and the flow rate is in the range of 0.011 kg/s ≤ Q ≤0.0113 kg/s . P and Q are rounded to 0.4 MPa and 0.011 kg/s , respectively.

### 2.2 Establishment of the weed-herbicide simulation model

#### 2.2.1 Weed model

Diverse weed species grow in wheat fields, including the commonly seen *Bromus japonicus* Thunb, *Alopecurus aequalis* Sobol., *Amaranthus retroflexus* L., and *Capsella bursa-pastoris* (Xu et al., 2023). Therein, *A. retroflexus* L. has wide leaves and therefore is very likely to affect the photosynthesis of wheat. Here, *A. retroflexus* L. was used as the prototype to establish a weed simulation model. Pro/Engineer (Pro/E) software can rapidly and accurately establish a three-dimensional (3D) model of products according to parameters including the size and shape of actual products and has become one of the main modeling software at present (Hongyu and Ziyi, 2011). The weed model was established in Pro/E 6.0. The research mainly studies the deformation of weeds under impact force in the herbicide application process, so geometric and mechanical parameters of leaves are mainly considered when establishing the model.

**Figure 3:**
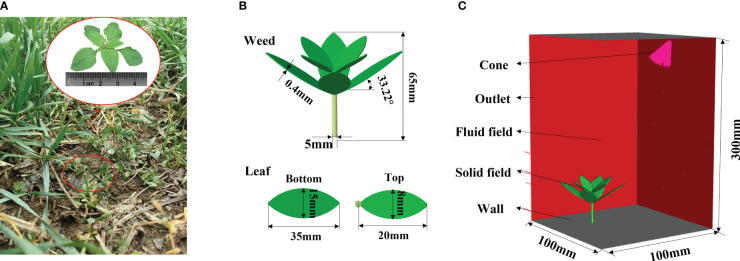
Simulation model. [**(A)** Measurement of geometric parameters of a weed; **(B)** establishment of the weed model; **(C)** setting of the fluid field model].

Weed plants were sampled in the wheat field of a test base in Nanjing Agricultural University (NJAU). Geometric parameters including the leaf angle and plant height were statistically analyzed (**Figure 3A**). All parameters were manually measured. Five weed plants in same type were randomly selected in the plot to summarize information including the plant height, leaf length, maximum width, and location of the maximum width, and average values of various parameters were calculated, as listed in **Table 1**. The leaves of *A. retroflexus* L. are ovoid or elliptic. They are entire or corrugated leaves arranged opposite on the stem (Ranlin and Sheng, 2006). The weed generally has four top leaves, and leaves continue to grow in the lower layer with the growth of the plant, forming a clearly layered structure. Leaves in various layers are staggered, and the stem is upright and thick. Two layers of leaves were modeled, the growth directions of top and bottom leaves had an angle of 45°, and the leaves were widest in the middle. The stem was modeled as a vertically growing cylinder. Finally, a 3D weed model was established in Pro/E, as displayed in **Figure 3B**.

**Table 1:** Basic parameters of the weed simulation model.

Geometric parameter	Value
Weed height (mm)	35
Number of leaves	12
Leaf angle (°)	33.222
Leaf thickness (mm)	0.4
Length of bottom leaves (mm)	35
Length of top leaves (mm)	20
Maximum width of bottom leaves (mm)	15
Maximum width of top leaves (mm)	8
Stem diameter (mm)	3
Mechanical parameter [29]	Value
Leaf density (kg/m³)	700
Elastic modulus (MPa)	222.85

Dynamic simulations of the weed were performed in the finite element analysis software ANSYS. As weed leaves are bent and deformed due to the herbicide spray, the transient structure of each leaf needs to be calculated. The leaves and stems are irregular geometries during mesh generation. To ensure the geometric adaptability and calculation accuracy of grids, tetrahedral grids of second-order elements were used for mesh generation of the weed plant. Therein, two layers of grids were set in the thickness direction of leaves to improve the calculation accuracy of deformation. The weed plant was set to be a fluid-solid interface (FSI), to which herbicide was applied to generate stress and calculate the leaf deformation under such stress.

#### 2.2.2 Fluid field model

In the fluid field analysis of the weed leaves, the computational domain measured 100 mm × 100 mm × 300 mm (**Figure 3C**). A cone was selected to represent the nozzle in the model, which was set obliquely above the weed according to Section 2.1, at the height of 300 mm, and its axis was aligned with the center of top leaves of the weed with *γ* of 35°. The top and bottom of the computational domain were both set as walls, and other outer surfaces were set as fluid outlets. The grid size in the computational domain was 200 mm. Due to the complex shape of fluid fields at the near-wall position, grids on each wall were refined to 2.5 mm, so that grid size showed smooth transition. The spraying pressure, flow rate, fluid density, and viscosity were 0.4 MPa, 0.011 kg/s, 1.225 kg/m³, and 1.7894 × $10^{-5}$ kg/ms, respectively. After naming the wall in contact with the plant as FSI, the liquid film formed by herbicide spray at the position and the stress could be exported.

#### 2.2.3 Setting of boundary conditions in the simulation

The governing equations of the weed and external flow field in the research are shown in the individual control of the solid and fluid fields. Therein, the plant deformation is mainly determined by the non-linear dynamic properties of the upper surface of leaves under the fluid pressure; because the pressure generated by the fluid is time varying, transient analysis is needed for the weed. The implicit algorithm can be selected for solution and expressed as **Equation (11)**.


(11) $$\left[M]\{\ddot{s}\}+[C]\{\dot{s}\}+[K]\{s\}=\{F\right\}$$

where [*M*] is the mass matrix of the weed plant; [C] represents the damping matrix of the weed plant; [*K*] is the stiffness matrix of the weed plant; {F} is the applied load vector of the weed plant; $\left\{\ddot{s}\right\}$ denotes the acceleration vector at grid nodes of the weed plant; $\left\{\dot{s}\right\}$ is the velocity vector at grid nodes of the weed plant; and {s} is the displacement vector at grid nodes of the weed plant.

The fluid in the research is formed by liquid particles formed by a transient discrete particle model (DPM). All liquid particles enter the fluid field via the upper surface of the computational domain along the fixed trajectory at the beginning of simulations. These discrete phase particles of these droplets can be used to calculate the motion trajectory using the force balance equation, as **Equation (12)**:


(12) $${\text{m}}_{\text{p}}\frac{{\text{d}v}_{\text{p}}}{\text{d}t}{\text{=m}}_{\text{p}}\frac{{\text{v}}_{\text{c}}{\text{-v}}_{\text{p}}}{{\text{t}}_{\text{r}}}{\text{+m}}_{\text{p}}\frac{{\text{g(ρ}}_{\text{p}}{\text{-ρ}}_{\text{c}})}{{\text{ρ}}_{\text{p}}}\text{+F}$$

where ${\text{m}}_{\text{p}}$ is the mass of liquid particles; ${\text{v}}_{\text{p}}$ stands for the jet velocity of liquid particles; ${\text{v}}_{\text{c}}$ is the velocity of the continuous phase; ${\text{t}}_{\text{r}}$ is the relaxation time of the liquid particle; g is the acceleration of gravity, namely, 9.8 m/ ${\text{s}}^{2}{\text{; ρ}}_{\text{p}}$ is the density of liquid particles; and F is the additional force in the fluid field.

The pressure of jet source is 0.4 MPa, so ${\text{v}}_{\text{p}}$ is much greater than that in the surrounding airflow velocity and apparent turbulence may be formed. The standard k–ϵ turbulence model that is highly robust in fluid computation is used to solve turbulences in the fluid field. When the herbicide spray reaches the plant wall, it mainly transfers pressure by forming a wall film, and the value of pressure is directly proportional to the wall thickness. The governing equation of the wall film is **Equation (13)**.


(13) $${\text{h=h}}_{0}+\frac{{\text{x}}^{2}{\text{+y}}^{2}}{2\text{R}}\text{+v(x, y)}$$

where *h* stands for the thickness of wall films at the weed plant wall; ${\text{h}}_{0}$ denotes the central film thickness, which is determined according to the load balance condition; *R* is the equivalent radius of curvature; and v is the velocity component of the herbicide spray in contact with the wall in different directions.

#### 2.2.4 Dynamic simulations of herbicide application

In the weed–herbicide joint simulation process, the FSI algorithm was used for solution, so as to obtain the interaction between weed structure and herbicide spray. In the structural solver, the mechanical properties of weed leaves were set, followed by mesh generation of the plant to discretize the model. In addition, the preset algorithm was used to solve the weed. In the fluid solver, the physical properties of liquid were set and grids in the fluid field were generated. The implicit algorithm was adopted to ascertain the state of the fluid in different regions. The two parts of calculation data are imported into the system coupling solver to allow interaction of calculation results on the FSI interface in each time step. Moreover, the displacement of weed leaves and the external flow field distribution of the nozzle were real-timely updated, with the coupling simulation time and the time step separately set to 0.5 s and 0.001 s.

**Figure 4:**
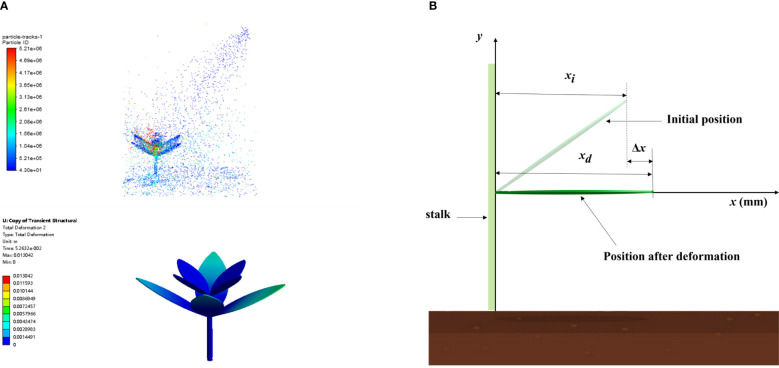
Dynamic simulations of herbicide application and calculation index. [**(A)** Dynamic simulations of the weed–herbicide interaction; **(B)** Schematic diagram for the calculation method of the weed leaf deformation index].


Dynamic simulations of herbicide application are shown in **Figure 4A**, which mainly summarize the weed leaf deformation and herbicide spray deposition. When calculating the weed leaf deformation, a coordinate system was constructed taking the horizontal direction of the leaf root as the x-axis, the extension direction of leaves as the positive direction of the x-axis, and the direction of growth of the stem as the positive direction of the y-axis (**Figure 4B**). The deformation index ξ is used to show leaf deformation, as **Equation (14)**.

(14) $$\text{ξ=}\frac{\text{Δx}}{{\text{x}}_{\text{i}}}$$

where Δx is the increment in the length of a leaf along the x-axis (mm), ${\text{Δx=x}}_{\text{d}}{\text{-x}}_{\text{i}}{\text{; x}}_{\text{i}}$ is the initial length of leaves along the x-axis (mm), and ${\text{x}}_{\text{d}}$ is the length of the deformed leaves along the x-axis (mm).

During computation of herbicide spray deposition, the ground area sheltered by the leaves is set as the test area, whereas areas in both sides are set as the control areas. Different areas share the same size in the direction of leaf width, all set to 15 mm. Because the original length of bottom leaves along the x-axis is 29.28 mm, it is rounded to 30 mm. The spray coverage in different areas is calculated using the **Equation (15)**:


(15) $$\text{η=}\frac{{\text{A}}_{\text{l}}}{{\text{A}}_{\text{t}}}\times 100\%$$

where ${\text{A}}_{\text{l}}$ is the area of the liquid film after spraying the herbicide; ${\text{A}}_{\text{t}}$ is the total area under test.

### 2.3 Field tests of application quality of herbicide

To assess the herbicide rate of utilization and weed leaf deformation under the selected operating parameters of the nozzle, tests were conducted on application quality of herbicide during the elongation stage in the wheat field in the test base of NJAU. Wheat in the test field was sown in drill and the test area was 5 m long.

**Figure 5:**
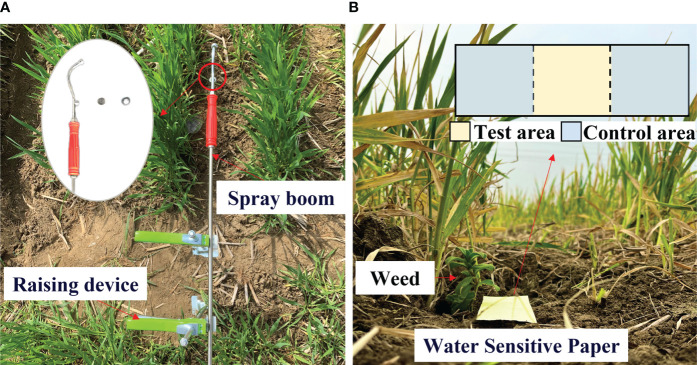
The layout of the field test. [**(A)** Layout of test devices; **(B)** Design of field tests].

Five positions were selected randomly as test points in the test area, and the tests were conducted using a small hand-push sprayer. Therein, a D3/DC33 (TeeJet Technologies) nozzle core was used and an electric diaphragm pump was adopted to adjust the spraying pressure, which remained at 0.4 MPa, and the flow rate was 0.011 kg/s. The spray boom was fixed on a raising device, as illustrated in **Figure 5A**, and the spray time in a single test was set to 1 s.

In the field tests on herbicide application, the weed leaf deformation under impact force during the action of herbicide spray was calculated using the equations and methods in Section 2.2.4. Image processing software Photoshop was used to extract images of leaves in different operating stages, and the growth direction is defined as the x-axis. The ruler tool was used to compute the leaf deformation index. The larger the ξ , the greater the deformation of the leaf. Water-sensitive paper was adopted to record the spray coverage (**Figure 5B**). The paper was placed below the weed and the center of the paper sheltered by the weed is the test area, whereas areas in both sides are control areas. The image processing method was used to calculate spray coverage in different areas below the weed using the following formula:


(16) $$\text{η=}\frac{{\text{y}}_{\text{num}}}{{\text{t}}_{\text{num}}}\times 100\%$$

where ${\text{y}}_{\text{num}}$ is the number of blue pixels after applying herbicide; ${\text{t}}_{\text{num}}$ represents the total number of pixels on the water sensitive paper.

The larger the η in the test area, the less the herbicide retained by weed leaves and the more the herbicide passing through the leaves and reaching the soil below. The nozzle was kept at the fixed position mentioned in Section 2.1. To reveal the improvement arising from use of the selected spraying pressure on the spray coverage of the weed, a high spraying pressure of 1 MPa was always applied in crop and fluid tests for comparison.

### 2.4 Data analysis

Calculated values in Equation 16 are coverages of the test area and the control areas in both sides; the difference of the herbicide in different areas cannot be directly compared. Considering this, the variance ( σ ) was introduced to evaluate the data deviation between areas the calculation method is as shown in **Equation (17)**:


(17) $$\sigma =\frac{1}{3}[{\left(\eta_{t}-\bar{\eta }\right)}^{2}+{\left(\eta_{cl}-\bar{\eta }\right)}^{2}+{\left(\eta_{cr}-\bar{\eta }\right)}^{2}]$$

where ${\text{η}}_{\text{t}}$ is the coverage in the test area (%); ${\text{η}}_{\text{cl}}$ denotes the coverage in the left control area (%); ${\text{η}}_{\text{cr}}$ is the coverage in the right control area (%); and $\bar{\eta }$ is the average coverage (%).

σ represents the deviation of coverage in different areas. The larger σ is, the greater the difference in the coverage in the test and control areas, the more the herbicide retained by leaves, and the higher the rate of utilization of herbicide.

To assess the accuracy of the model, the normalized mean absolute error (NMAE) was adopted as the evaluation index of simulation results. The calculation method is as shown in **Equation (18)**:


(18) $$\text{NMAE=}\frac{1}{\text{n}}\sum \frac{{|\text{y}}_{\text{ti}}{\text{-y}}_{\text{simi}}|}{{\text{y}}_{\text{simi}}}$$

where *n* is the sample size; ${\text{y}}_{\text{ti }}$ is the test value at the ith point; and ${\text{y}}_{\text{simi}}$ is the predicted value of the model at the ith point.

NMAE represents the mean absolute error of predicted values of the model against the test values, and it can reflect the accuracy of the prediction model. Previous research has shown that a model can predict the test data if the NMAE is smaller than 30% (Saha et al., 2021). The lower the NMAE, the more accurate the prediction.

## 3 Results

### 3.1 Analysis of simulation results

#### 3.1.1 Deformation of weed leaves under impact force during the weed–herbicide interaction

**Figure 6:**
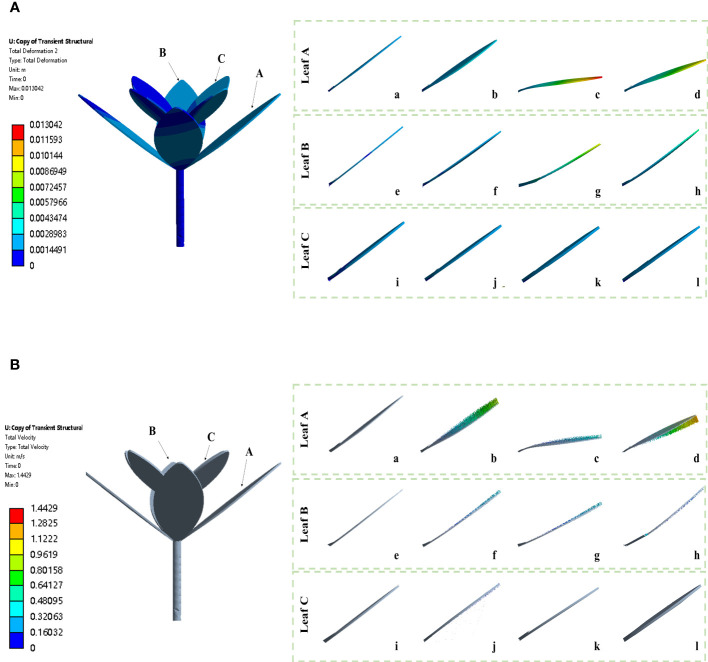
Weed leaf deformation and velocity vectors under impact force in the simulation tests. [**(A)** a-d, e-h, and i-l separately represent deformation of leaves A, B, and C under impact force in the targeted movement, contact and diffusion, continuous coverage, and strong impact and drainage stages; **(B)** a-d, e-h, and i-l separately represent vector velocity of leaves A, B, and C under impact force in the targeted movement, contact and diffusion, continuous coverage, and strong impact and drainage stages].

The weed plant is deformed to different extents under action of herbicide spray. To ascertain the deformation trend of weed leaves under impact force during the weed–herbicide interaction, the deformation and velocity vectors of the weed in different operating stages were exported (**Figures 6A, B**). The different contact positions between herbicide and weed leaves and different amounts of herbicide spray in the herbicide application process give rise to differences in leaf deformation. Considering this, three typical leaves (A, B, and C) at different positions of the weed were selected for detailed analysis. Leaves A, B, and C separately represent a bottom leaf near the nozzle, a bottom leaf far from the nozzle, and a top leaf. As shown in **Figure 6**, various leaves are slightly deformed under gravity in the targeted movement stage. The weed state in the stage can be defined as the initial state. After entering the contact and diffusion stage, leaf A is rapidly deformed downward from the original leaf angle (**Figure 6A**a) ξ is 0.084, and the amount of retained herbicide increases. In the continuous coverage stage, the leaf deformation reaches the maximum, ξ is 0.427, and the leaf is quasi-parallel to the ground, when the herbicide carried by the leaf reaches the peak. As herbicide application continues, the leaf is not further deformed downward. The velocity vector shows that the leaf has an upward velocity (**Figure 6B**d), and ξ is 0.298. Leaf B is also deformed downward after entering the contact and diffusion stage and ξ is 0.082 (**Figure 6A**f); in the continuous coverage stage, the leaf reaches the position with the maximum deformation (**Figure 6A**g) and ξ is 0.276, followed by continuous swinging of the leaf (**Figures 6A**h, **B**h), when ξ is 0.0692. Leaf C is at the top of the plant, where the velocity of leaf is generally lower than that of bottom leaves (**Figures 6A**ii–l) and the leaf only shows slight swinging after being struck by the herbicide spray (**Figures 6A**i–l).

**Figure 7:**
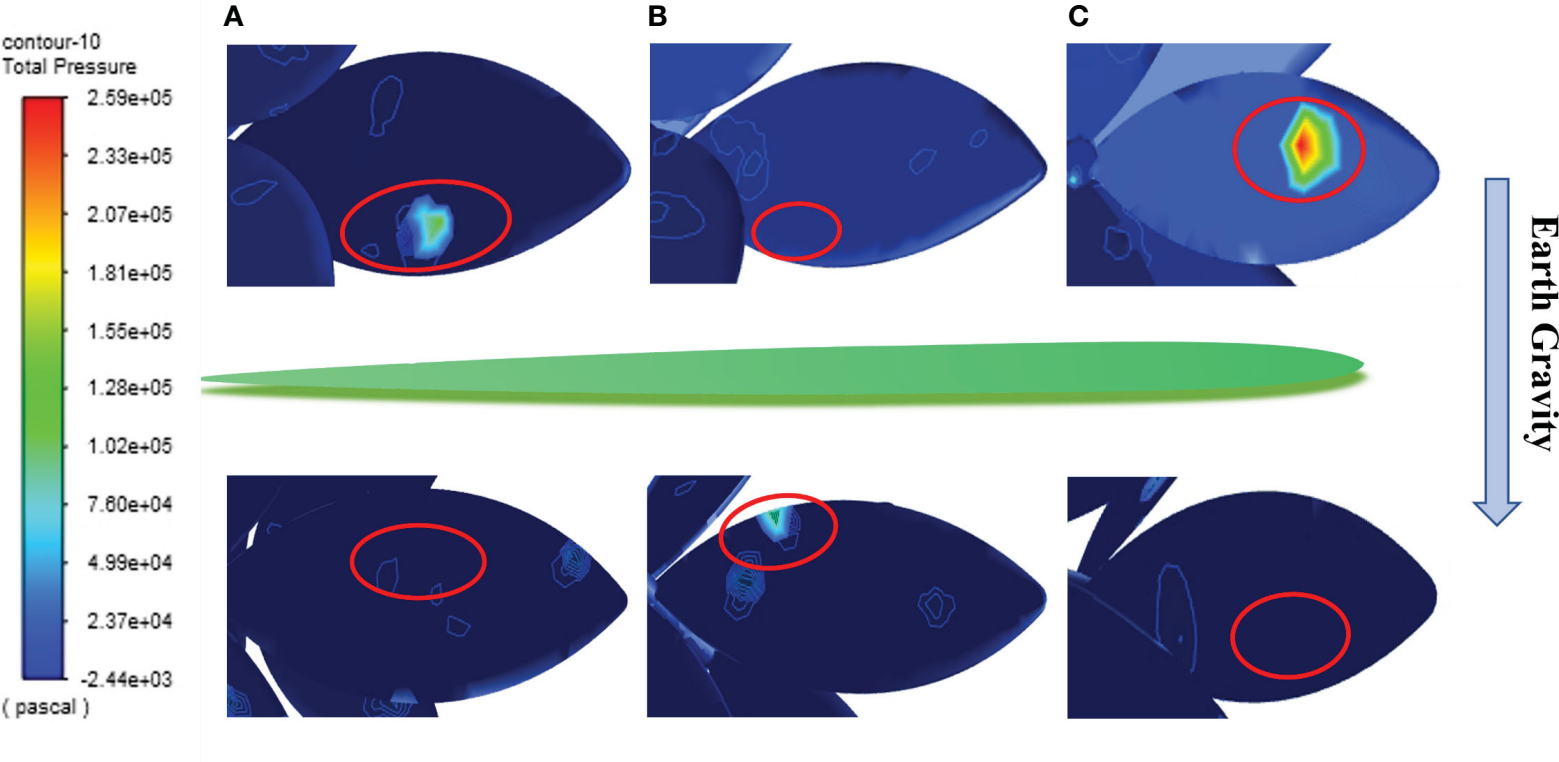
The pressure distribution on the upper and lower surfaces of leaves at different positions.(**A, B, C** represent the front and back surfaces of plant leaves **A, B,** and **C,** respectively. The red boxes indicate the locations of the highest pressure points on each leaf).

The pressure distribution on the weed under action of herbicide spray is exported (**Figure 7**). Analysis reveals that leaves are deformed mainly because of the significant pressure difference on the upper and lower surfaces of leaves due to herbicide spraying, so that leaves at various positions are deformed under impact force (A, B, and C in **Figure 7**). It is worth noting that leaves at different positions all swing with different amplitudes after reaching the maximum deformation. Combining with **Figures 6A, B**, weed leaves at different positions are deformed to different extents under the spraying pressure of 0.4 MPa. Moreover, the leaves show the maximum deformation when they are quasi-parallel to the ground under continuous action of herbicide spray, suggesting that herbicide application does not enter the strong impact and drainage stage. Therefore, the selected herbicide application parameters can block flow of the sprayed herbicide to the soil below, thus significantly solving the problem of a low herbicide adhesion rate occurring because herbicide passes through leaves during herbicide application.

#### 3.1.2 Flow direction of herbicide under the weed–herbicide interaction

**Figure 8:**
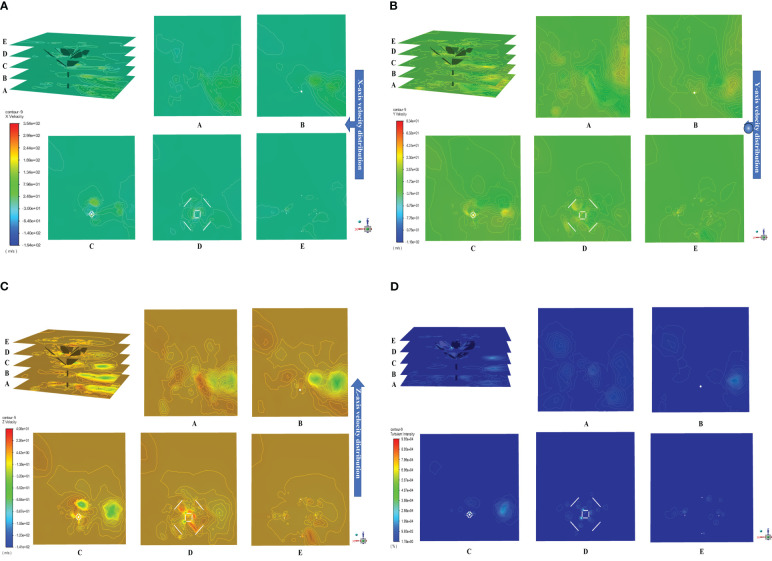
Distribution of external flow field. [**(A)** Velocity distribution in the *x*-direction; **(B)** Velocity distribution in the *y*-direction; **(C)** Velocity distribution in the *z*-direction; **(D)** Distribution of turbulence intensity in the external flow field].

After being ejected from the nozzle, the herbicide spray forms an external flow field in the space analyzed. Due to being sheltered by weed leaves, the external flow field is distributed non-uniformly. Statistical analysis was performed on the herbicide spray coverage in the sheltered area below leaves and areas in both sides. Through calculation using Equation 16, the spray coverage in the test area is 16%, whereas those in the control areas in both sides are 72% and 68%. The area of the liquid film below leaves is smaller than that in areas in both sides, implying that the herbicide spray can be retained by weed leaves. In the postprocessing process, a slicing tool was adopted to divide the fluid field; because the research focuses on the flow field of the weed plant, only the position of the weed was sliced with an interval of 8.5 mm, as shown in **Figure 8**. Plane A is the bottom of the weed plant. The velocity field distributions in the x-, y-, and z-directions in the fluid field were exported (**Figures 8A–C**). As shown in planes A~E, the velocity field is distributed non-uniformly after the herbicide spray reaches the weed position from the liquid inlet. The maximum fluid velocity in the x direction is found above the leaves (**Figure 8A** D) (7.27 m/s), the fluid velocity in the y direction is large at the top of the plant and above leaves (**Figures 8B** C,D,E) (maximum of 14.15 m/s), and the fluid velocity in the z-direction is large around the plant and above leaves (**Figure 8C** C) (maximum of 7.74 m/s). The fluid velocity is always large in areas above leaves in each direction. The velocities at spatial positions in **Figures 8A–C** are generally 0 m/s, which means that little herbicide is splashed to other positions. Meanwhile, the velocity below leaves in each direction is generally lower than that above leaves, indicating that the herbicide spray is mainly retained by leaves whereas it does not flow to the bottom.

The turbulence intensity reflects the eddies and vortices of herbicide spray formed in the external flow field. The stronger the turbulence intensity, the more violent the motion of eddies and the larger the velocity gradient of the fluid. **Figure 8D** illustrates the distribution of turbulence intensity in the external flow field. When the fluid encounters an obstruction, the fluid direction changes and forms turbulences, so the turbulence intensity near the positions of weed leaves is stronger than that elsewhere; because weed leaves are not bent to an excessive extent, the amount of herbicide retained by top leaves is much greater than that retained by leaves at other positions, so the turbulence intensity above the weed is higher (plane D). As the herbicide flows downward, the amount of herbicide retained and the kinetic energy both decrease, and some herbicide reaches the ground and induces turbulence (planes B and C) whereas the intensity is lower than that at the top. Given the complex structure of the weed plant, it is difficult to predict the spatial positions and overlapping of leaves and therefore the formation positions of turbulences and vortexes cannot be estimated. In comparison, in **Figure 8D**, the turbulence intensity in the middle and lower parts of the weed is 2%, which indicates that the fluid is steady in spatial flow under the spraying pressure of 0.4 MPa. Only a small amount of herbicide rebounds from the weed leaves, whereas most is retained thereon; therefore, the selected spraying pressure can improve the rate of utilization, and reduce waste, of herbicide due to flow to soil during weeding.

### 3.2 Field test results

#### 3.2.1 Spray coverage under the weed–herbicide interaction

**Figure 9:**
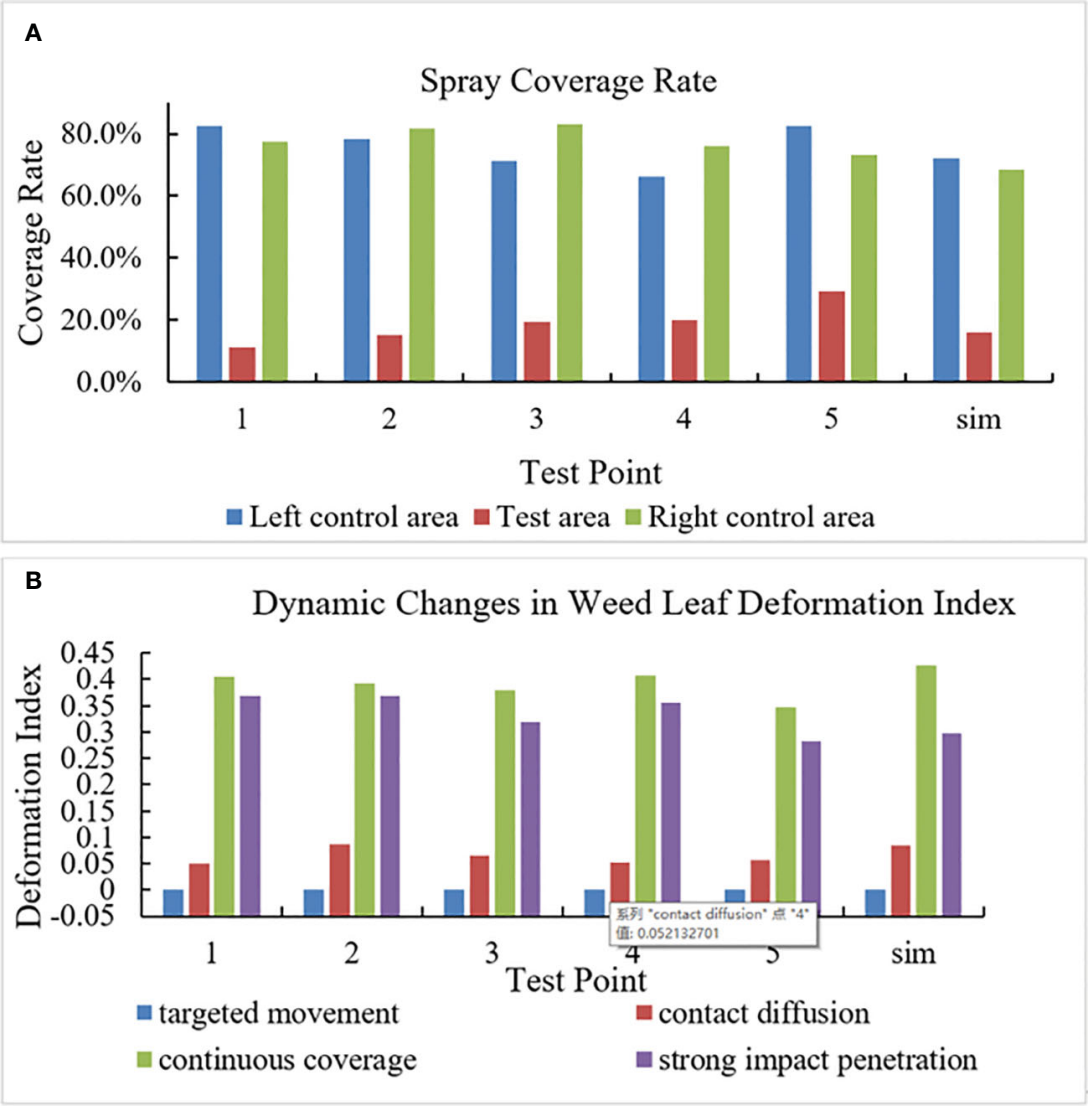
Field test data. [**(A)** Spray coverage at various test points; **(B)** Weed leaf deformation index at various test points].

The method in Section 2.3 was used to calculate the spray coverage η at different test positions of the water-sensitive paper. The larger the η is, the more the total deposition of herbicide spray on the paper. The spray coverage in each test area is shown in **Figure 9A**. Statistical analysis shows that under the spraying pressure of 0.4 MPa and the flow rate of 0.011 kg/s, σ is 5%~11%, which means that the spray coverage varies spatially. In particular, ${\text{η}}_{\text{t}}$ , ${\text{η}}_{\text{cl}}$ , and ${\text{η}}_{\text{cr}}$ are 11%~20%, 66%~82%, and 73%~83%, respectively. As the spraying pressure rises to 1.0 MPa, σ declines to 1%~3%, ${\text{η}}_{\text{t}}$ increases to 40%~55%, ${\text{η}}_{\text{cl}}$ is 72%~86%, and ${\text{η}}_{\text{cr}}$ is 68%~89%. The result indicates that the selected spraying pressure can increase the herbicide retention rate on weed leaves. The test and simulation values at various test points were compared, and the NMAE under each spraying pressure was calculated. Under 0.4 and 1.0 MPa, NMAEs are 15% and 11% in fluid tests, which are both lower than 30%, so the established fluid model can be used predict the fluid distribution ejected from the nozzle.

#### 3.2.2 Weed leaf deformation under impact force during the weed–herbicide interaction

In images collected in the simulation and field tests, the state in which the weed leaves have not contacted with the herbicide was designated as the initial position to calculate weed leaf deformation index ( ξ ) in different stages, as illustrated in **Figure 9B**. Because the plant leaves are not deformed in the targeted movement stage, NMAE in the stage is not discussed whereas it is 27%, 9%, and 16% in other stages. This indicates that the established weed simulation model is able to predict the dynamic changes in the weed.

**Figure 10:**
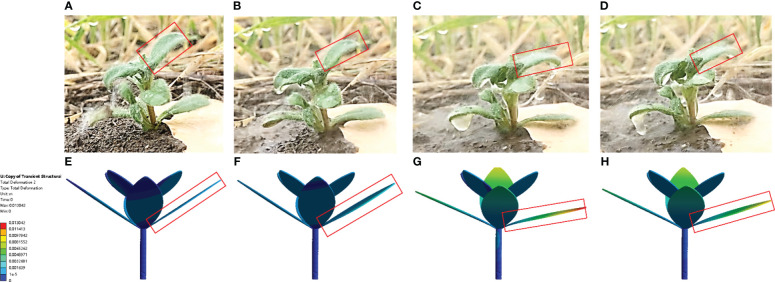
Weed leaf deformation in field and simulation tests [**(A–E)** Leaf deformation in field tests in different operating stages; **(F–J)** Leaf deformation in simulation tests in different operating stages].

The deformation images of leaves in different operating stages in the simulation and field tests were exported, as shown in **Figure 10**. The results show that leaves below the nozzle are not obviously bent downward to form a channel to reach the ground. Therefore, the selected spraying pressure can solve the problem of a low herbicide rate of utilization caused by herbicide passing through the weed. The dynamic leaf deformation predicted at different times remain consistent with that *in-situ*.

## 4 Discussion

The research on precision herbicide application should not only focus on realization of targeted herbicide spraying but also on ensuring adhesion of herbicide onto weed leaves. Previous research has shown that changing the operating parameters of the spraying system can improve the spray coverage in the operating area (Guler et al., 2007; Li et al., 2018). Lin et al. (Lin et al., 2022) studied herbicide spray deposition in the working area in the crop field under different spraying pressures and spray angles; the results show that changes in the spray angle only slightly affect the herbicide adhesion while increasing the spraying pressure can improve the herbicide adhesion rate in the target area. Guler et al. (Guler et al., 2012) compared the spray coverages under different flow rates at the nozzle (0.0126 kg/s, 0.019 kg/s, and 0.0378 kg/s) and two spray heights (0.5 m and 0.7 m), analysis shows that as the spraying height decreases, the spray coverage is significantly larger than that under other combinations of parameter. In comparison, these studies mainly adjust the spraying system pressure and flow rate according to the spray coverage and by reducing the spray drift rate; however, they ignore the bearing capacity of leaves and therefore their ability to retain the herbicide, so that most herbicide flows to the soil, leading to a low rate of utilization and waste. The closer the nozzle to the middle of wheat rows is, the higher the spraying precision while the higher the difficulty of operation. Therefore, the nozzle was fixed at the middle between rows and the height was set as the height of commonly seen weeds.

Starting from analysis of the weed–herbicide interaction, the research explored weed leaf deformation under impact force in the herbicide application process and established the dynamic model of weed leaves. In this way, appropriate operating parameters including the spraying pressure and flow rate of the spraying system were determined, and the herbicide adhesion rate on the weed leaves is improved (Li et al., 2018). Simulation tests can analyze the dynamic weed–herbicide interaction process (Endalew et al., 2009; Hong et al., 2018). To study the dynamic deformation of weed leaves and flow direction of herbicide under the weed–herbicide interaction, the dynamic simulation model of herbicide application was built using the finite element method. The results show that the herbicide spray generated by the nozzle with the selected operating parameters all does not cause the leaves to deform more than those parallel to the ground at different times. This indicates that the herbicide is mainly retained by the plant, which significantly improves the spray coverage on the weed during operation of the nozzle.

The velocity vector shows that the bottom leaf has an upward velocity (**Figure 6B**d), arising because the leaf swings slightly after reaching equilibrium. The top leaves of the plant are deformed to a negligible extent, this is because the leaf in the area is shorter than the bottom leaves and is therefore stiffer, so that the leaf swings less than those in other areas, and suggesting that only a small amount of herbicide passes through the top leaves. Each disturbed leaf undergoes “frequency locking,” which leads to swinging of different amplitudes and is consistent with research results (Gosselin and de Langre, 2009). The velocity distribution in the external flow field of the nozzle is mainly concentrated around the weed, which means that not too much herbicide is splashed elsewhere. The fluid velocity is large at the position above the top leaves; this is because after coming into contact with top leaves of the weed, some herbicide spray retains a certain kinetic energy and therefore rebounded due to the elasticity of leaves (Hu et al., 2022). This part of herbicide spray accumulates at the top of the weed. Due to the adhesion and shear action of leaves for herbicide spray, the original flow state is broken and vortexes are formed (Finnigan, 2010).

The turbulence intensity is low in the external flow field on the whole, which indicates that the herbicide flows steadily in those areas near the weed; therefore, the selected operating parameters of the nozzle can improve the herbicide rate of utilization (Py et al., 2006). Field tests on application quality of herbicide were conducted in the test base of NJAU, results show that the nozzle with the selected operating parameters does not cause large deformation of weed leaves during herbicide application, which solves the problem of herbicide waste because the herbicide passes through the weed (He et al., 2018). The simulation test results share the consistent trend with the field test results, and the NMAE is smaller than 30%, this suggests that the established dynamic simulation model for operation of the nozzle can be used to predict dynamic changes in the weed plant and herbicide during weeding (Saha et al., 2021).

The herbicide spraying speed is a key factor that determines the direction of herbicide flow after coming into contact with weed leaves (Hu et al., 2022). Limited by the existing test devices, data of the key factor were not collected in the field tests. A specific test bench should be built to measure and record the herbicide spraying speed. When establishing the finite element model, different fluids feature different physical parameters, which may change the distribution of fluids in the external flow field and the amounts of fluid adhered to, and rebounding from, weeds (Ellis et al., 1997). Physical parameters of aqueous herbicide solution were adopted in the established dynamic simulation model of herbicide application, whereas influences of physical parameters of different herbicides on the disturbance and spray coverage of weed plants were not discussed. The interaction model of herbicides commonly used in wheat fields and weeds should be established in future research to ascertain the external flow field distribution and the weed leaf deformation during herbicide application.

## 5 Conclusion

1) A dynamic model for impact force of droplets on leaves was established, and the suitable operating parameters of the spraying system were determined. The deformed leaves are parallel to the ground, resulting in the amount of retained herbicide reaching the maximum under spraying pressure of 0.4 MPa and flow rate of 0.011 kg/s when the spray height is 300 mm.
2) The results of the dynamic simulation of pesticide application indicate that under suitable operational parameters, the deformation index of weed leaves ranges from 0.08 to 0.43. The degree of leaf deformation is inversely proportional to the spraying distance, with the maximum deformation occurring at the tip of the leaves. The external flow field is distributed non-uniformly, the velocities at spatial positions are generally 0 m/s, little herbicide is splashed to other positions, and the velocity below leaves in each direction is generally lower than that above leaves, indicating that the herbicide spray is mainly retained by leaves.
3) The results of field test show that under suitable operational parameters, the maximum leaf deformation index is 0.41 and the average spray coverage in the sheltered area below the leaves is 19%, whereas those in the control areas in both sides are separately 76% and 78%, the herbicide has a good retention rate on weed leaves, and the herbicide coverage on weed leaves has increased by 31% compared with that under a spraying pressure of 1.0 MPa, thus achieving precision herbicide application to weeds in wheat fields.

When applying spray, factors such as wind speed and temperature in the air also need to be considered. To demonstrate the universality of the selected operational parameters for the spraying system, future work can focus on studying the distribution of the herbicide in the presence of wind disturbance.

## Data availability statement

The raw data supporting the conclusions of this article will be made available by the authors, without undue reservation.

## Author contributions

QX: Conceptualization, Data curation, Formal analysis, Investigation, Methodology, Software, Visualization, Writing – original draft, Writing – review & editing. MS: Formal analysis, Investigation, Validation, Writing – original draft. TW: Investigation, Software, Validation, Writing – original draft. WC: Resources, Supervision, Writing – review & editing. YZ: Resources, Supervision, Conceptualization, Writing – review & editing. JN: Conceptualization, Funding acquisition, Methodology, Project administration, Writing – review & editing.
